# Night lizards survived the Cretaceous–Palaeogene mass extinction near the asteroid impact

**DOI:** 10.1098/rsbl.2025.0157

**Published:** 2025-06-25

**Authors:** Chase D. Brownstein, Saúl F. Domínguez-Guerrero, José D. L. Tufiño, Martha M. Muñoz, Thomas J. Near

**Affiliations:** ^1^ Department of Ecology and Evolutionary Biology, Yale University, New Haven, CT, USA; ^2^ Ciudad Universitaria, Coyoacán, Mexico; ^3^ Departamento de Biología Evolutiva, Ciudad Universitaria, Coyoacán, Mexico

**Keywords:** lizards, Cretaceous–Palaeogene, asteroid, phylogenetics, extinction, life history, physiology

## Abstract

Discovering traits that facilitate survival through mass extinctions is of great interest to scholars of macroevolution. Here, we show that the common ancestry of xantusiid night lizards, a species-poor lineage with conserved anatomy, precedes the Cretaceous–Palaeogene boundary. We reconstruct the geographic distribution of the stem lineages of three living genera, *Lepidophyma, Xantusia,* and the monospecific *Cricosaura*, as surrounding the Gulf of Mexico, identifying *Xantusiidae* as the only tetrapod crown clade currently known to have survived the Cretaceous–Palaeogene mass extinction proximal to the site of the bolide impact on the Yucatan Peninsula. By integrating data from living species, we show that the night lizard lineages that witnessed the bolide impact likely possessed low litter or clutch sizes of 1–2 individuals, challenging prevailing hypotheses derived from studies focused on species-rich clades such as birds and mammals about what traits are necessary to survive a terrestrial mass extinction.

## 1. Introduction

Mass extinctions are a fundamental force of change on the macroevolutionary timescale [1–6], and the investigation of the biological variables that have modulated persistence through mass extinction has become an interdisciplinary effort uniting palaeontology [7–15] and evolutionary biology [16–20]. Among Earth’s mass extinctions, the Cretaceous–Palaeogene (K–Pg) Mass Extinction, which occurred 66.02 Ma after an asteroid struck what is now the Yucatan Peninsula of Mexico [21], stands out for its importance to the construction of present-day communities [7,22,23]. Although the long-term effects of this mass extinction in shaping the global biota have been extensively investigated, recent work has focused on understanding the immediate fallout of the asteroid impact and whether factors such as the proximity of ecosystems to the impact event [24–29], the timing of year [30] and the interactions within ecological communities [10,31] affected direct biotic recovery. It is unclear whether any vertebrate clades endemic to the region surrounding the impact event—North America, Central America and the Caribbean—are old enough to be K–Pg survivors [32–34].

Xantusiid night lizards (*Squamata*: *Xantusiidae*) and solenodons (*Mammalia*: *Solenodontidae*) may be the only clades that have persisted in the Gulf of Mexico region since the Mesozoic era [32–36]. Yet, recent work supports a Cenozoic crown age for *Solenodontidae* [34,35]. Xantusiids are diurnal-crepuscular lizards [37] notable for their microhabitat specialization [32,37–39], slow metabolisms [40], microendemic species [32,41,42], and historically puzzling phylogenetic relationships [33]. Using a Bayesian tip-dating protocol using DNA sequences from seven nuclear genes, we demonstrate that the crown age of *Xantusiidae* predates the K–Pg extinction. Our findings indicate that night lizards have been endemic to North and Central America since the earliest Late Cretaceous. Intriguingly, our research reveals that ancestral night lizards ancestrally possessed very small litter sizes, challenging conventional wisdom about life history traits associated with survival through catastrophic events. The fact that night lizards endured the K–Pg extinction while inhabiting regions surrounding the impact site further complicates our understanding of extinction survival mechanisms.

## 2. Methods

### (a) Sequence dataset assembly and maximum likelihood phylogenetic analysis

We combined all published nuclear gene data for xantusiids by building on the dataset of Noonan *et al.* [32] using sequences available on GenBank for seven genes: *aenolase*, *bdnf*, *cmos*, *gapd*, *nt2*, *pomc* and *rag1*. We excluded mitochondrial sequences because of their capacity to artificially inflate divergence time estimates [43]. Using the IQ-TREE webserver, we conducted maximum likelihood analyses of the nuclear gene sequence dataset and assessed nodal support using ultrafast bootstrap supports and approximate Shimodaira–Hasegawa (SH) tests.

### (b) Time calibration

We inferred a time-calibrated phylogeny for *Xantusiidae* using a Bayesian tip-dating approach implemented in the program BEAST v. 2.6.6 [44,45] on the concatenated nuclear gene sequences. We placed fossils on the tree based on the phylogenies presented in Brownstein *et al.* [46], Gauthier *et al.* [47] and Schatzinger [48], but let the positions of the two included species of †*Palaeoxantusia* remain unfixed relative to one another given the likely paraphyly of this genus [48]. The ages of included species also follow Brownstein *et al.* [46]. Although one possible pan-xantusiid, †*Retinosaurus hkamtiensis*, is known from the Cretaceous of southeastern Asia, we excluded this taxon from our tip-dating analyses because its relationships among scincoids remain unstable [49]. We used a general time reversible (GTR) model of nucleotide evolution, a relaxed lognormal clock for the clock model, and the BEAST2 implementation of the fossilized birth–death model [50]. We set the diversification rate at 0.03 following [51] and with the following input values: 34 living species of xantusiids, median origin prior of 165.3 Ma (the age of the near-crown stem squamate †*Bellairsia gracilis*) [52]. We set the bounds of the origin prior to 161.5 Ma, the base of the Bathonian, which is the approximate lower bound of the age of crown *Squamata* estimated in phylogenetic analyses of genome-wide marker data [53,54], and 251.9 Ma, the Permian–Triassic boundary, before which no crown lepidosaur fossils are currently known [55–57]. We ran BEAST2 twice independently over 100 million generations and a 10 million pre-burnin, checked for convergence of the posteriors and effective sample size values over 200 in Tracer v. 1.7.1 [58], combined the top 75% of trees using LogCombiner v. 2.6.6., and summarized them in a single maximum clade credibility with median node heights in TreeAnnotator v. 2.6.6.

### (c) Ancestral state reconstruction and historical biogeography

We reconstructed historical biogeography along our tip-dated phylogeny using the R package BioGeoBears [59]. We binned sampled tips into six regions (Africa and Madagascar; Cuba; North America including the Baja California peninsula; Central America, consisting of the rest of Mexico to Panama; Channel Islands, to account for the island endemic *Xantusia riversiana*), ran historical biogeography reconstruction using three models (dispersal-extinction cladogenesis, DEC; dispersal-vicariance, DIVALIKE; Bayesian biogeographic model, BAYAREALIKE) with and without the addition of a jump dispersal parameter (+j), and compared model fit using Akaike Information Criterion (AIC) scores. Lastly, we conducted ancestral state reconstruction of litter size in the R package phytools [60] using the fastAnc method on litter size data we collected from the literature and from examination of museum specimens (electronic supplementary material). *Cricosaura typica* is oviparous [39,61] , so the more accurate term for this species is clutch size. However, we used the term ‘litter size’ throughout the manuscript for simplicity and because all species of *Xantusia* and *Lepidophyma* are viviparous.

## 3. Results and discussion

Our phylogenetic analyses place the Cuban night lizard *Cricosaura typica* as the sister species to all other night lizards with robust nodal support and resolve relationships among the recently diversified living species of *Xantusia* and *Lepidophyma* with moderate nodal support, as in previous analyses [32,33,41,42,49]. Our tip-dated Bayesian phylogeny places the most recent common ancestor of living xantusiids deep within the Cretaceous, 92.62 Ma (95% HPD: 74, 110.94 Ma), slightly older than the 81 Ma age for *Xantusiidae* estimated by Noonan *et al.* [32] and considerably older than the ages inferred by Vicario *et al.* [33], which range from 43.07 to 64.93 Ma.

**Figure 1.**
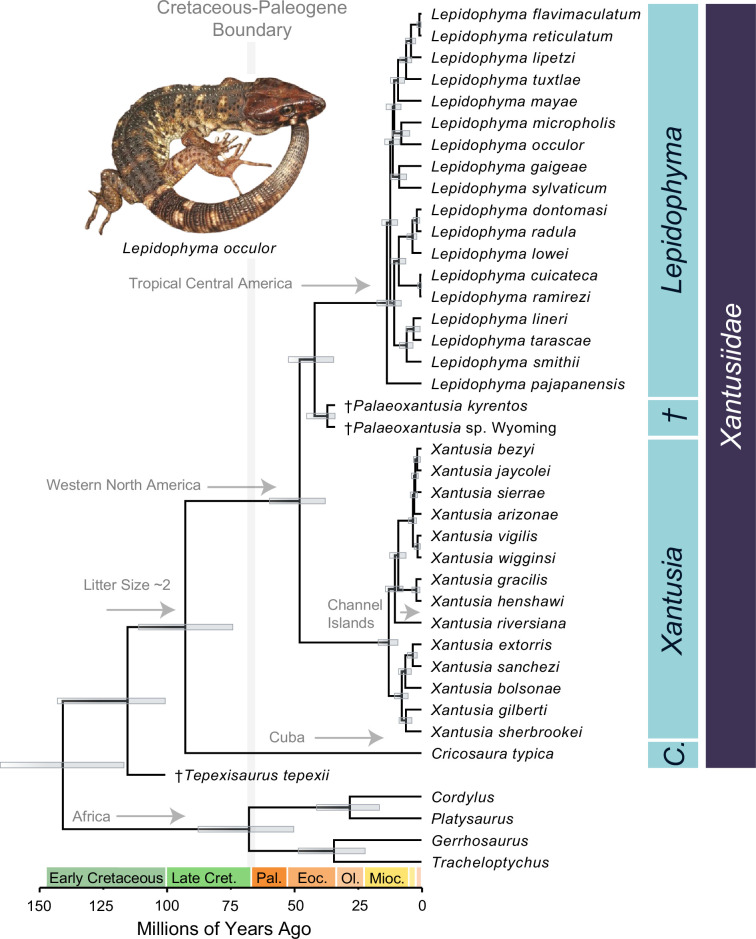
Night lizards survived the K–Pg mass extinction proximal to the impact site. Time-calibrated phylogeny of *Xantusiidae* resulting from the BEAST tip-dating analysis, with scincoid outgroups shown. Bars at nodes indicate 95% highest posterior density (HPD) intervals. Daggers (†) indicate extinct taxa. Annotations at nodes indicate biogeographic and life history state shifts. Photograph of *Lepidophyma occulor* by José Daniel Lara Tufiño, used with permission. Abbreviations: Late Cret., Late Cretaceous; Pal., Palaeocene; Eoc., Eocene; Ol., Oligocene; Mioc., Miocene.

We estimate that crown lineage *Lepidophyma* and *Xantusia* originated recently and nearly simultaneously in Central and North America at 12.26 Ma (95% HPD: 10.26, 17.46 Ma) and 12.83 Ma (9.24, 16.98 Ma), respectively. Because we infer that the giant Channel Islands xantusiid *X. riversiana* (island night lizard) is nested within other living species of *Xantusia* rather than representing the living sister as in a previous molecular phylogeny [32], our analyses support a recent dispersal of this taxon to the isolated Channel Islands and acquisition of gigantism. Our analyses place the divergence between *X. riversiana* and its sister lineage among members of *Xantusia* at 10.54 million years ago (95% HPD: 7.24, 14.16 Ma). Given that the Channel Islands originated in the last 5 million years [62,63] (with ephemeral connectivity to the mainland via shallow water during glacials) [64,65], we infer that dispersal to these islands occurred along the branch leading to the island night lizard (figure 1).

The results of our tip-dating analysis support the hypothesis that at least two lineages of xantusiids persisted through the K–Pg extinction event. This finding, combined with our historical biogeographic reconstruction and observations of the fossil record [33,47,48,66], suggests that xantusiids have continuously inhabited North–Central America since the Mesozoic era. Notably, these lizards would have occupied ecosystems that directly surrounded the asteroid impact site. This makes *Xantusiidae* unique among living families of terrestrial vertebrates, as they are the only known survivors of the K–Pg mass extinction in close proximity to the impact location. Consequently, xantusiids present an excellent opportunity to study ecological and life history traits that may have contributed to their survival. Xantusiid lizards possess a number of intriguing ecological, physiological and life history traits, including low fecundity [67], low resting metabolic rates [40,68] and specialized microhabitat preferences [32,32,38,69,70]. Ancestral state reconstruction of litter size in xantusiids suggests the most recent common ancestor of crown xantusiids had a litter size of approximately two (figure 1; electronic supplementary material, figure S2). Uncertainty in the ancestral litter size estimate (95% CI on ancestral state: −2.22835239, 6.846803) is likely driven by the limited species diversity of xantusiids; we were only able to sample some of the living species diversity of this already species-poor clade. Our estimate falls between the single egg laid by *C. typica*, the only oviparous xantusiid, and the larger litter sizes (2–6) observed in the island night lizard [39,67]. As expected given their association across squamate diversity [71–73], there is also a clear association between litter size and body size in xantusiids, as larger litter sizes are observed in the island night lizard and large-bodied species of *Lepidophyma* (electronic supplementary material, figure S2). Together, these observations suggest that larger clutch sizes in *X. riversiana* and some *Lepidophyma* likely represent the derived condition among xantusiids.

The survival and subsequent diversification of mammals and birds following the K–Pg mass extinction [16,74–79] have led to the expectation that traits such as increased offspring number might confer resilience during extinction events [16]. Xantusiid night lizards, however, present an intriguing contrast to these assumptions. While they align with some expected traits—our inference that *X. riversiana* resulted from recent dispersal suggests that, like crown birds [17,17,18,79,80] and placental mammals [31,81,82], the xantusiid lineages that crossed the K–Pg boundary were small-bodied—they diverge in other aspects. Notably, the potential persistence of xantusiids through the K–Pg as narrow-ranging [41,42,69] species with small offspring numbers challenges classic assumptions about the natural history of extinction-surviving clades [83,84]. These observations of xantusiid biology are particularly interesting given that range size is universally considered a crucial factor in survival through mass extinctions [4,6,85,86]. The survival of xantusiids through the K–Pg boundary, despite their specialized habitat requirements and low reproductive output, presents a fascinating case study of chance survival through biological catastrophe. This apparent contradiction between the biology of xantusiids and their survival highlights the complexity of factors influencing extinction resilience. It suggests that our understanding of what enables species to persist through major extinction events may need to be refined, with the xantusiid example offering valuable insights into the diverse strategies that can lead to evolutionary persistence.

## Data Availability

All data are available in the supplementary material for this article [87].
